# Total knee arthroplasty in patients with severe obesity: outcomes of standard keeled tibial components versus stemmed universal base plates

**DOI:** 10.1186/s43019-023-00184-4

**Published:** 2023-04-11

**Authors:** Katherine L. Elcock, Deborah J. MacDonald, Nick D. Clement, Chloe E. H. Scott

**Affiliations:** 1 University of Edinburgh Medical School, Chancellor’s Building, The Royal Infirmary of Edinburgh, 49 Little France Crescent, Old Dalkeith Road, Edinburgh, EH16 4SB UK; 2 Edinburgh Orthopaedics, Royal Infirmary of Edinburgh, Edinburgh, UK; 3 Department of Orthopaedics, University of Edinburgh, Edinburgh, UK

**Keywords:** Total knee arthroplasty, Obesity, Tibial baseplate, Aseptic loosening, Varus alignment

## Abstract

**Background:**

Patients with severe obesity [body mass index (BMI) ≥ 40 kg/m^2^] potentially overload the tibial component after total knee arthroplasty (TKA), risking tibial subsidence. Using a cemented single-radius cruciate-retaining TKA design, this study compared the outcomes of two tibial baseplate geometries in patients with BMI ≥ 40 kg/m^2^: standard keeled (SK) or universal base plate (UBP), which incorporates a stem.

**Methods:**

This was a retrospective, single-centre cohort study with minimum 2 years follow-up of 111 TKA patients with BMI ≥ 40 kg/m^2^: mean age 62.2 ± 8.0 (44–87) years, mean BMI 44.3 ± 4.6 (40–65.7) kg/m^2^ and 82 (73.9%) females. Perioperative complications, reoperations, alignment and patient-reported outcomes (PROMS): EQ-5D, Oxford Knee Score (OKS), Visual Analogue Scale (VAS) pain score and satisfaction were collected preoperatively, and at 1 year and final follow-up postoperatively.

**Results:**

Mean follow-up was 4.9 years. SK tibial baseplates were performed in 57 and UBP in 54. There were no significant differences in baseline patient characteristics, post-operative alignment, post-operative PROMs, reoperations or revisions between the groups. Three early failures requiring revision occurred: two septic failures in the UBP group and one early tibial loosening in the SK group. Five-year Kaplan–Meier survival for the endpoint mechanical tibial failure was SK 98.1 [94.4–100 95% confidence interval (CI)] and UBP 100% (*p* = 0.391). Overall varus alignment of the limb (*p* = 0.005) or the tibial component (*p* = 0.031) was significantly associated with revision and return to theatre.

**Conclusions:**

At early to mid-term follow-up, no significant differences in outcomes were found between standard and UBP tibial components in patients with BMI ≥ 40 kg/m^2^. Varus alignment of either tibial component or the limb was associated with revision and return to theatre.

## Highlights

- Favourable knee arthroplasty outcomes in BMI ≥ 40 kg/m^2^ with different tibial baseplates.
- No overt advantage of UBP over standard keeled tibia in patients with BMI≥40kg/m2, though may be underpowered to demonstrate this. 
- Varus alignment of tibial component or limb associated with increased revision rate.
- Further research needed to investigate theoretical benefit of longer tibial stem.

## Introduction

Total knee arthroplasty (TKA) is an effective treatment for knee osteoarthritis. Severe obesity, defined as a body mass index (BMI) ≥ 40 kg/m^2^, is a risk factor for developing osteoarthritis and for requiring TKA [1, 2]. Though complication rates following TKA are higher in patients with increased BMIs [3], TKA remains cost effective in this patient group [4], with no difference in patient-reported outcome measure improvement [4] or survival [5] compared with patients with normal BMIs. Though improved polyethylene engineering has led to an overall reduction in aseptic loosening [6, 7], it remains an important mode of TKA failure [8]. There remains significant concern that overload in severely obese patients may result in early tibial subsidence or loosening [9, 10] and some authors have recommended the routine use of additional tibial stem fixation in patients with high BMIs [10–14].

Complex kinematics at the knee with rollback and rotation during flexion in addition to six degrees of freedom create numerous forces that TKA implants must resist, including compression, tension, axial torque, varus/valgus moments and shear [9]. To reduce shear, micromotion and tibial lift-off, projections of different geometries are added to the under-surface of tibial components including keels, stems or pegs [9]. Whilst stems provide stability, they introduce their own shear forces and as they are load bearing, stress shielding of metaphyseal bone occurs along their length [9, 15, 16]. In turn, stress shielding potentially increases the risk of tibial subsidence, loosening and fracture.

**Fig. 1:**
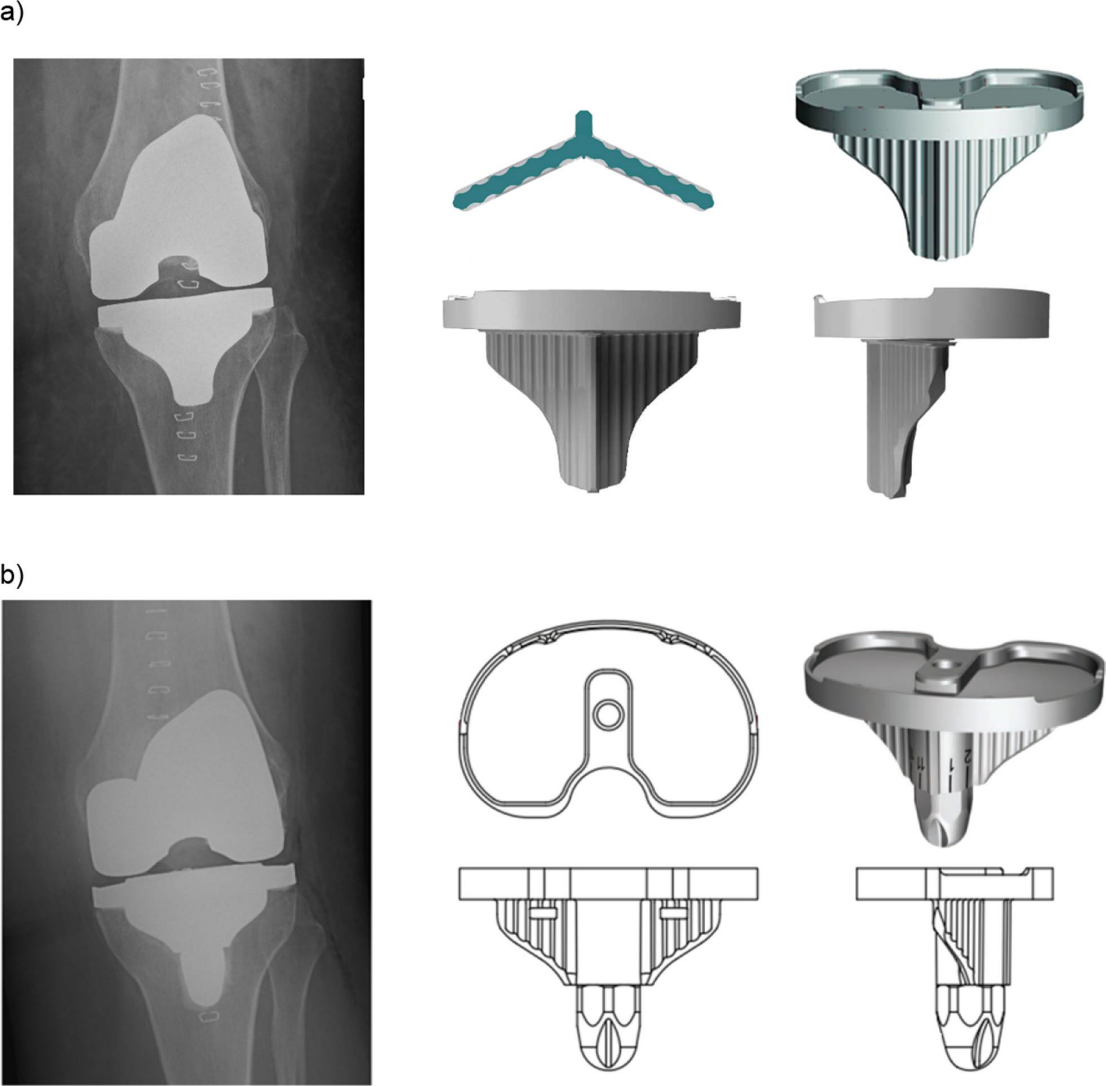
Tibial component designs and radiographic appearance: (**a**) standard keeled tibial component geometry and (**b**) universal base plate geometry incorporating a short stem

Standard stemless tibial component geometry varies considerably across TKA implants. Though most incorporate a short stem and keels as part of tibial baseplate design, the lengths of these projections vary. The standard keeled tibial baseplate of the Triathlon (Stryker, Mahwah, NJ) TKA consists of anti-torque keels for rotational stability without any stem element (Fig. 1a). Both keel thickness (2.6–3.6 mm) and keel depth (28–39 mm) vary across sizes in SK tibial components. An alternative baseplate, the universal base plate (UBP), is available as part of the system and incorporates a 20 mm keel and a boss/stem of 40 mm depth and 16 mm diameter to which additional stems can be added if desired (Fig. 1b). These dimensions are consistent across all sizes (Fig. 1a, b). Both SK and UBP tibial components are made from cobalt chrome.

The aim of this study was to compare the outcome of the cemented standard keeled tibial baseplate (SK) with the cemented UBPs in severely obese patients (BMI ≥ 40 kg/m^2^) undergoing TKA. The outcomes of interest were early mechanical failure of the tibial component requiring revision, patient-reported outcomes (PROMs), pain, complications, reoperation and all-cause revision.

## Materials and methods

Ethical approval was obtained for this retrospective cohort study (Scotland (A) Research Ethics Committee 16/SS/0026). Following review of a prospectively collected, single-centre arthroplasty database, 111 patients with a BMI ≥ 40 kg/m^2^ undergoing unilateral Triathlon (Stryker, Mahwah, NJ, USA) TKA for end-stage degenerative joint disease by eight surgeons at a large orthopaedic teaching hospital from 2013 to 2018 were included. The second of bilateral TKAs was excluded.

All patients underwent primary cemented cruciate retaining Triathlon TKAs via a medial parapatellar approach. Patient demographics were recorded. Standard keeled tibial components (SK) and universal base plates (UBPs) were available for all cases and were selected at the surgeons’ discretion. Electronic patient records were reviewed for complications (early, ≤ 30 days and late, > 30 days), reoperations and revision surgeries.

Prior to TKA and at 1 year following surgery, patients completed postal questionnaires including comorbidity scoring and validated patient-reported outcome measures (PROMs): the EuroQol 5-dimension score (EQ-5D) [17], the Oxford Knee Score (OKS) [18], Visual Analogue Scale (VAS) pain scores (0–100), and satisfaction was measured at 1 year using a five-point Likert scale from ‘very dissatisfied’ to ‘very satisfied’ [19]. The EQ-5D is a validated and widely used five-dimension multi-attribute general health questionnaire that defines an overall health index (from −0.594 to 1). The OKS is a validated knee-specific outcome measure, in which 12 questions (five possible answers) give scores from 0 to 48 (higher scores = better function). A minimal important change (MIC) in OKS was defined as seven points [20]. Patients recorded the presence or absence of 18 comorbidities as recorded by the Charlson Comorbidity Index (CCI): myocardial infarction, heart failure, peripheral arterial disease, cerebrovascular disease, dementia, chronic obstructive pulmonary disease, connective tissue disorder, peptic ulcer, diabetes, kidney disease, hemiplegic stroke, leukaemia, malignant lymphoma, solid tumour, liver disease, acquired immunodeficiency syndrome, back pain, pain from other joints and hypertension [21].

Overall femorotibial alignment (femorotibial angle, FTA), and tibial component alignment in coronal (medial proximal tibial angle, MPTA) and sagittal (posterior tibial slope, PTS) planes were measured on short leg weight-bearing radiographs by the senior author using the method described by Sarmah et al. [22]. An MPTA < 87° was defined as a varus tibia and a femorotibial angle < 177° was defined as varus lower limb alignment.

Follow-up PROMs questionnaires were resent to all patients in January 2021. In addition to the OKS, VAS-pain score, EQ-5D and patient satisfaction scores, patients were asked if they had undergone any reoperations to the operated knee and to select any areas of the knee (as many as applied) that were painful from the following: the front of the knee, the back of the knee, the inside edge, the outside edge, the top of the shin bone, all around the knee or other, with a free text box.

### Statistical analysis

Data were analysed using SPSS version 25.0. Variables were tested for normality. Univariate analysis was performed using parametric (unpaired Student’s *t*-test) and non-parametric (Mann–Whitney *U*-test) tests as appropriate to assess differences in continuous variables between SK and UBP cohorts. Nominal categorical variables were assessed using Chi squared or Fisher’s exact tests. Pearson’s correlation was used to correlate continuous variables. Survival analysis was undertaken with Kaplan–Meier analysis using the endpoints any revision, mechanical failure and any reoperation. The log rank statistic was used to compare the survival of treatment strategies. A *p*-value < 0.05 was considered statistically significant.

## Results

Over the study period, 111 patients met the inclusion and exclusion criteria: mean age was 62.2 [standard deviation (SD) 8.0, range 44–87] years; mean BMI 44.3 (SD 4.6, range 40–65.7) kg/m^2^ and 82 (73.9%) were female. Of the 111, 57 underwent TKA with an SK tibial baseplate and 54 with a UBP. Mean length of follow-up was 4.9 years (SD 1.7 years) with a minimum of 2.1 years. There were no differences in baseline patient characteristics (Table 1) or comorbidities between standard and UBP groups (*p* > 0.05).

**Table 1:** Pre-operative patient characteristics

Variable	Standard (*n* = 57)	UBP (*n* = 54)	Mean difference (95% CI)	*p*-Value
Age (years)	64.8 (8.0)	63.6 (8.0)	−1.23 (−4.2 to 1.8)	0.903*
Female sex	40 [70]	42 [78]		0.392^
BMI in kg/m^2^	42.5 (40.6–43.9)	43.4 (41.4–46.4)		0.234**
Number of comorbidities	1 (0–1)	1 (0–2)		0.656**
Indication				
OA	55 [96]	54 [100]		0.388^
IA	1 [2]	0		
Post-HTO	1 [2]	0		
Length of hospital stay (median, days)	5 (4–6)	4 (3–6)		0.049**
Length of F/U (years)	5.1 (1.6)	4.6 (1.8)	−0.50 (−1.1 to 0.1)	0.496*
Pre-operative PROMs				
EQ-5D VAS	65.3 (20.4)	63.8 (22.7)	−1.5 (−9.6 to 6.6)	0.296*
EQ-5D	0.296 (0.31)	0.271 (0.31)	−0.03 (−0.141 to 0.09)	0.723**
Pain VAS	49.8 (23.2)	49.4 (20.3)	−0.41 (−8.7 to 7.9)	0.226*
OKS	17.4 (7.3)	17.6 (7.1)	0.27 (−2.5 to 3.0)	0.594*

Mean (SD), median (IQR), number [%]. ^Chi square, *t-test, **Mann–Whitney *U*-test

### Alignment

**Fig. 2:**
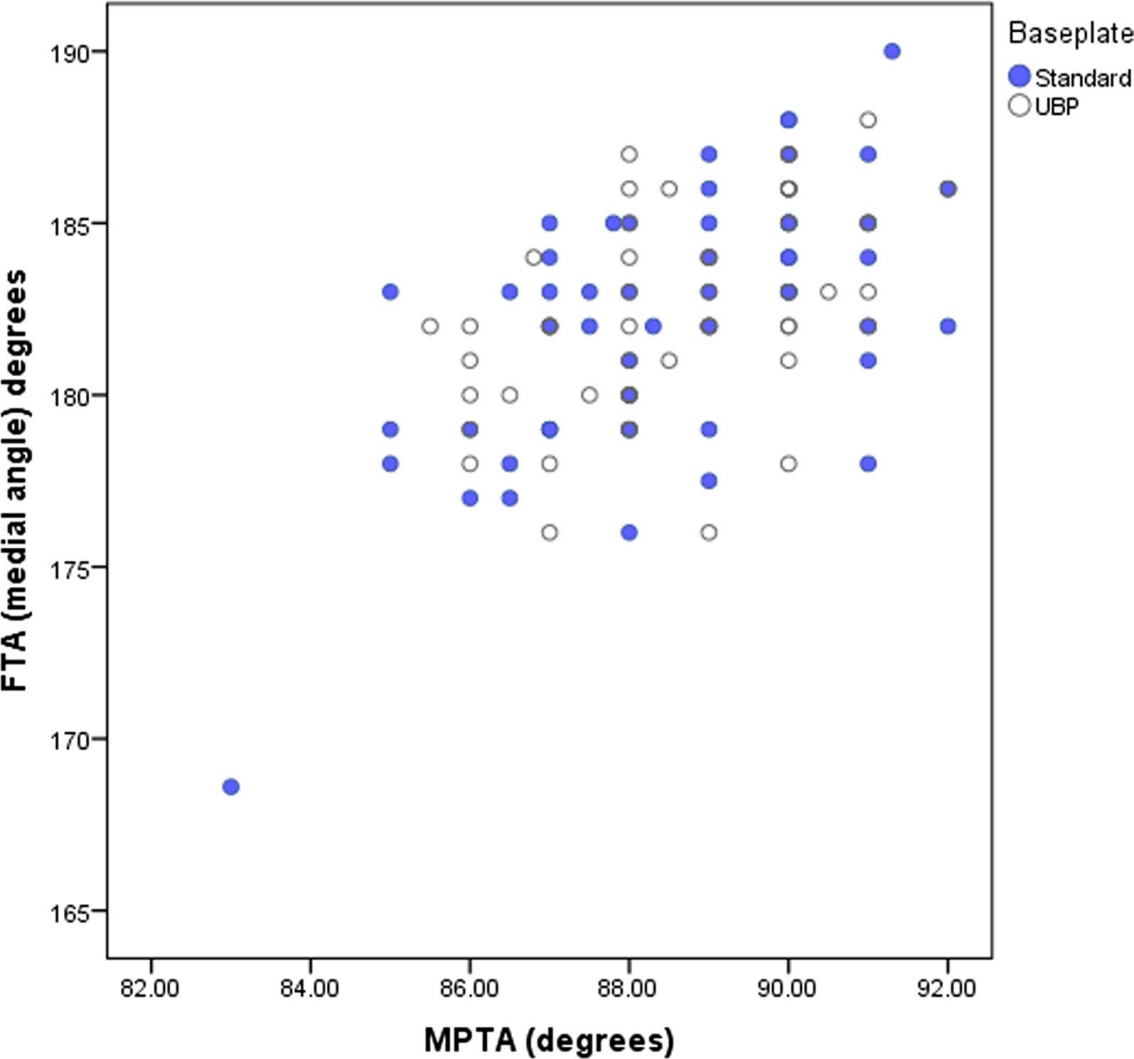
Coronal plane alignment: medial proximal tibial angle (MPTA) and medial femorotibial angle (< 180° = varus)

There were no significant differences in the mean tibial component alignment achieved after TKA or in overall femorotibial alignment (FTA) between tibial baseplate groups (Table 2), nor were there differences in the number of varus or valgus outliers (Fig. 2).

**Table 2:** Tibial component and limb alignment in degrees

Variable	Standard (*n* = 58)	UBP (*n* = 54)	Mean difference (95% CI)	*p*-Value
FTA	182.3 (3.7)	182.4 (2.9)	0.14 (−1.1 to 1.4)	0.822*
MPTA	88.6 (2.0)	88.6 (1.6)	−0.005 (−0.7 to 0.7)	0.986*
PTS	2.6 (3.0)	3.4 (2.9)	0.81 (−0.3 to 1.9)	0.157*

Femorotibial angle (FTA) is medial (< 180° = varus). *MPTA* medial proximal tibial angle, *PTS* posterior tibial slope

* indicates t-test

### PROMs

**Fig. 3:**
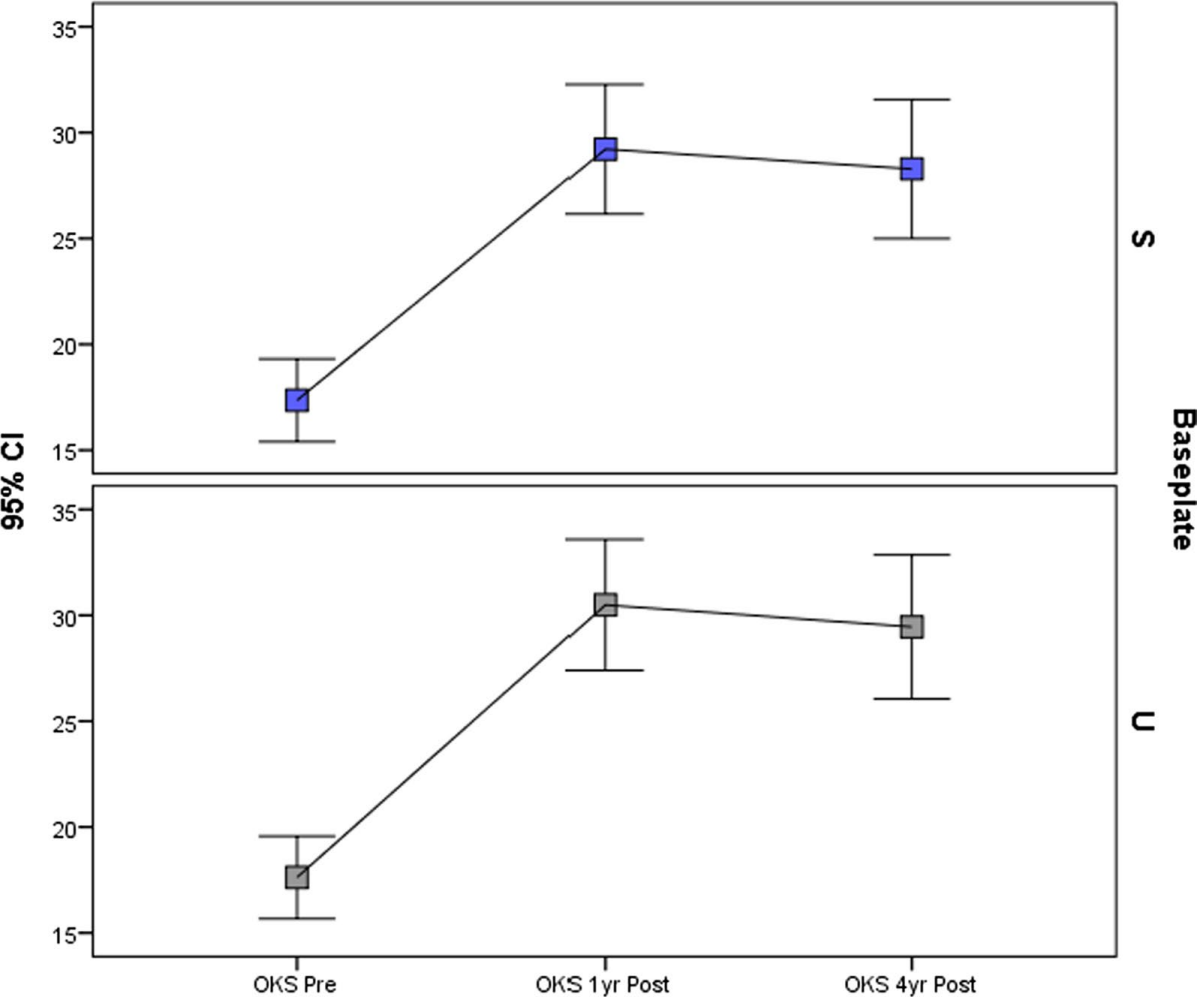
OKS according to tibial baseplate design over the study period. *S* standard keeled tibial baseplate, *U* universal base plate

**Fig. 4:**
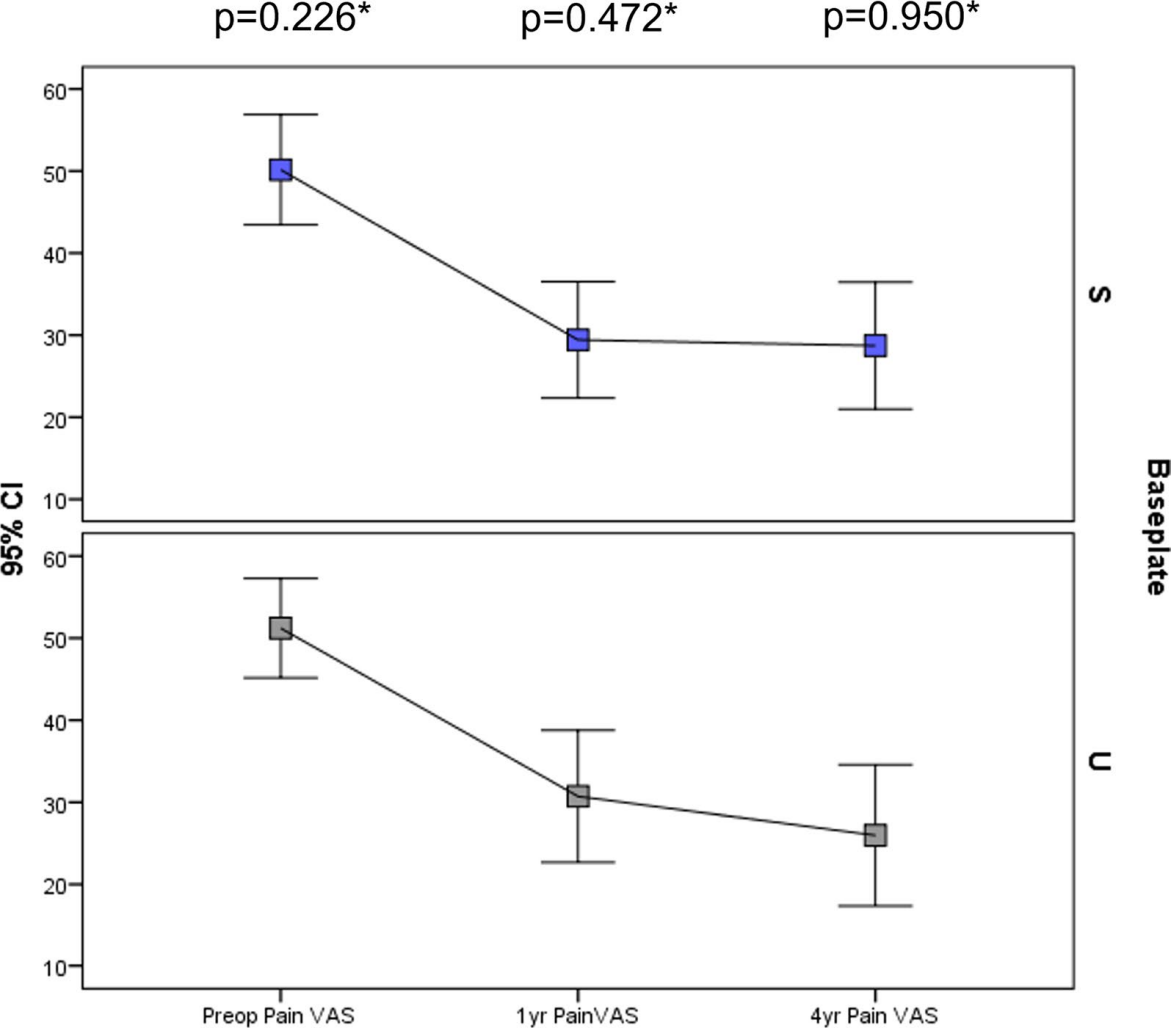
Visual analogue pain scales at each timepoint according to tibial baseplate design. *S* standard keeled tibial baseplate, *U* universal base plate

**Fig. 5:**
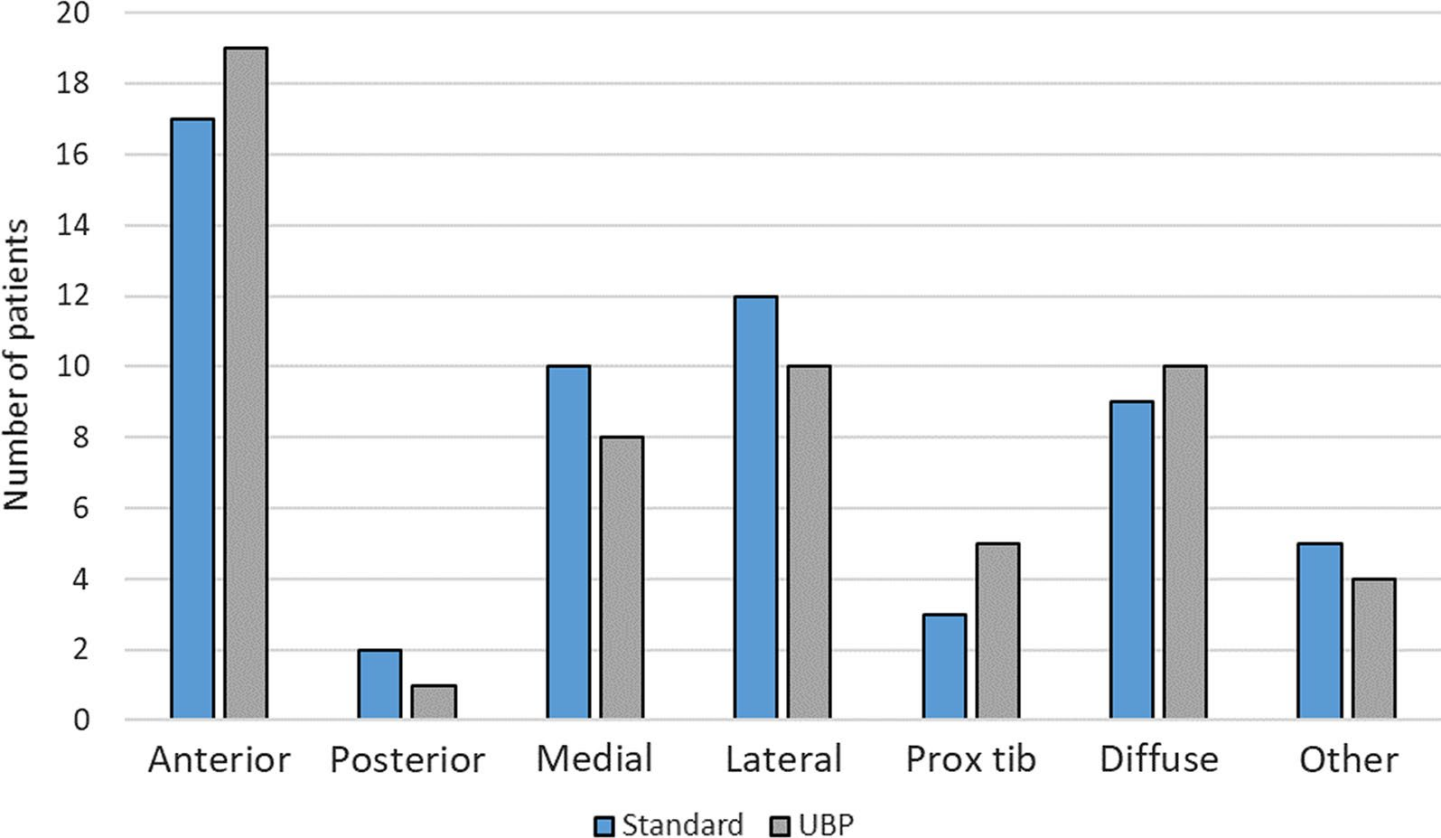
Pain location according to tibial base plate design. No significant differences in pain distribution were found

**Fig. 6:**
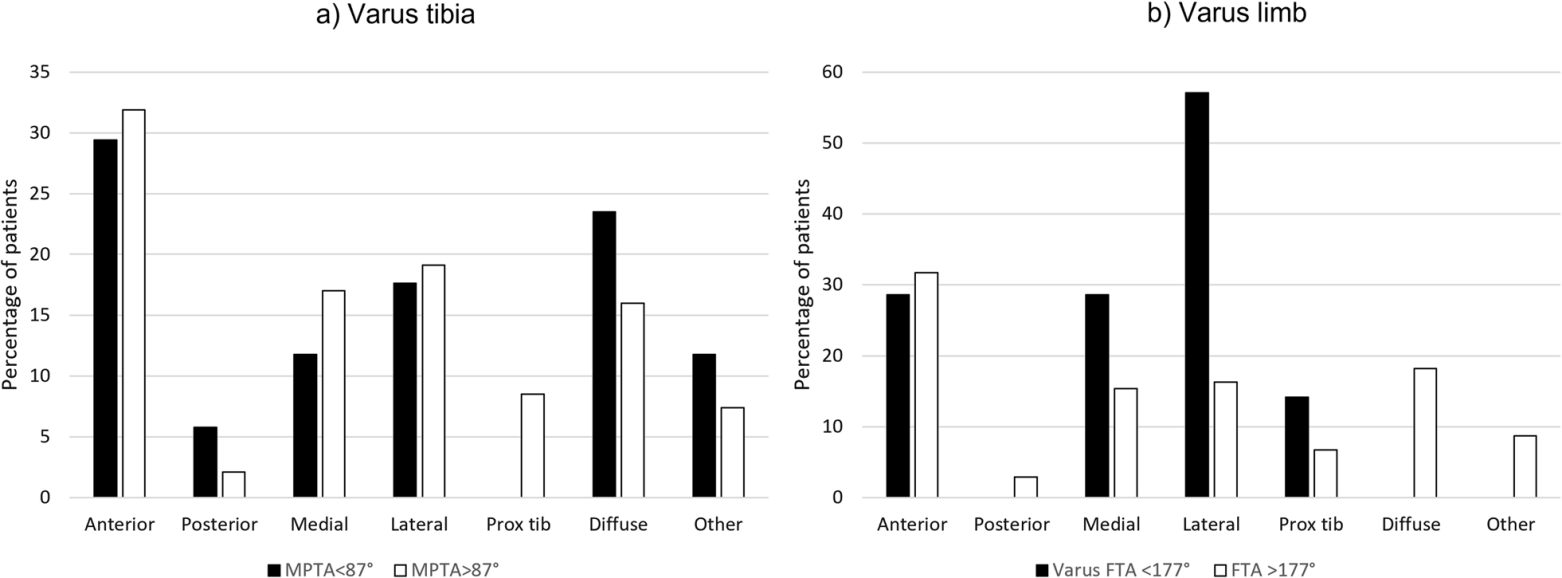
Proportion of patients reporting pain by anatomic location according to (**a**) varus medial proximal tibial angle (MPTA) < 87° and (**b**) varus femorotibial angle < 177°. Lateral pain was more common in varus aligned limbs (*p* = 0.023 Fisher’s exact test)

There were no statistically significant differences in improvement in OKS, EQ-5D or pain scores at 1 year or at final follow-up (Table 3; Figs. 3, 4). Pain location did not differ significantly between tibial baseplate designs (*p* > 0.05, Chi square, Fig. 5). A varus limb alignment with FTA of < 177° (i.e. > 3° varus) was associated with lateral knee pain (*p* = 0.023 Fisher’s exact test, Fig. 6) but did not significantly affect the prevalence of pain in any other region of the knee (*p* > 0.05). A varus tibia with MPTA of < 87° did not significantly affect pain location (*p* > 0.05, Fig. 6). For the entire cohort, a varus FTA of < 177° was significantly associated with revision (2/5 versus 1/104, *p* = 0.010 Fisher’s exact test) and reoperation (3/7 versus 4/104 *p* = 0.005 Fisher’s exact test), with a relative risk of revision of 14.4 (4.46–46.5 95% CI) and a relative risk (RR) of any reoperation of 11.1 (3.1–40.3 95% CI). In contrast, a varus MPTA < 87 alone was not significantly associated with revision (1/17 versus 2/94, *p* = 0.396 Fisher’s exact test) or reoperation (1/17 versus 6/94, *p* = 1.0 Fisher’s exact test). The study was under-powered to detect a similar trend for mechanical failure.

**Table 3:** Post-operative patient-reported outcome measures

Variable	Standard (*n* = 58)	UBP (*n* = 54)	Mean difference (95%CI)	*p*-Value
Improvement in				
VAS pain at 1 year	16.6 (32.0)	20.2 (32.6)	3.6 (−8.6 to 15.8)	0.490*
VAS Pain at 4 years	21.1 (37.0)	22.8 (36.3)	1.7 (−13.6 to 17.0)	0.822*
EQ-5D at 1 year	0.321 (0.34)	0.352 (0.37)	0.03 (−0.10 to 0.17)	0.672*
EQ-5D at 4 years	0.296 (0.36)	0.291 (0.38)	−0.005 (−0.15 to 0.13)	0.769*
OKS at 1 year	11.9 (10.1)	12.9 (10.8)	1.01 (−3.0 to 5.0)	0.524*
OKS at 4 years	10.9 (11.8)	11.8 (12.3)	0.90 (−3.7 to 5.5)	0.760*
1 year satisfaction				0.928^
Very satisfied	24 [41]	26 [48]		
Satisfied	23 [40]	18 [33]		
Neutral	7 [12]	6 [11]		
Dissatisfied	2 [3]	2 [4]		
Very dissatisfied	2 [3]	1 [2]		
Final follow-up satisfaction				0.561^
Very satisfied	24 [41]	24 [44]		
Satisfied	19 [33]	14 [26]		
Neutral	6 [10]	7 [13]		
Dissatisfied	3 [5]	6 [11]		
Very dissatisfied	5 [9]	2 [4]		

*Unpaired *t*-test, ^Chi Square

### Complications and revisions

Overall, there were no statistically significant differences in early or late complications (Table 4) or reoperations between baseplate designs.

**Table 4:** Complications

Early complications	Standard (*n* = 58)	UBP (*n* = 54)	*p*-Value
< 6 weeks			0.410^
AF	0	1	
Pneumonia	2	1	
VTE	4	1	
Cellulitis	3	3	
Wound complication	7	5	

There was one case of early tibial loosening/subsidence requiring revision. It occurred at 35 months after TKA with a SK baseplate in a 74-year-old female patient with a BMI of 43 kg/m^2^ and lymphoedema with a tibial alignment of MPTA of 83° and a subsequent FTA of 169°. Two patients from the UBP group underwent revision for infection. Four patients returned to theatre: 2/58 of the SK baseplate group for manipulation under anaesthetic (MUA), and 2/54 of the UBP group where one MUA and one wound washout and closure for dehiscence were performed (*p* = 0.710 Fisher’s exact test).

**Fig. 7:**
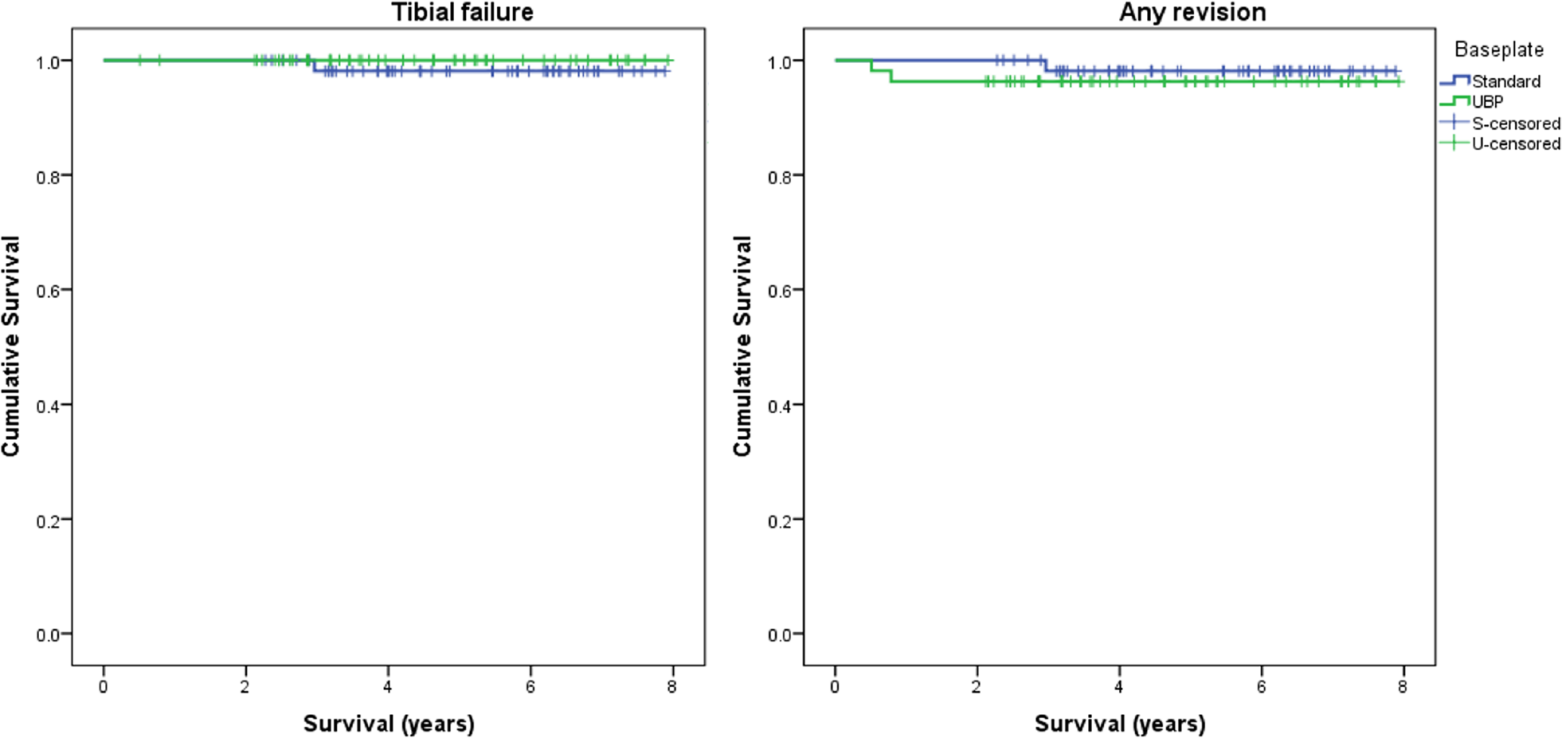
Kaplan–Meier survival graphs for the endpoints (**a**) tibial failure and (**b**) any revision

Kaplan–Meier estimates of survival and life tables are provided in Tables 5 and 6 and Fig. 7. There were no significant differences in 5 year survival between tibial baseplate designs for the endpoints any reoperation, any revision or tibial failure.

**Table 5:** Five-year Kaplan–Meier survival estimates with log rank statistic

	Five-year survival (95% CI)		
	Standard	UBP	*p*-Value
Any reoperation	94.7 (88.8–100)	92.6 (85.5–99.7)	0.588
Any revision	98.1 (94.4–100)	96.3 (91.1–100)	0.475
Tibial failure	98.1 (94.4–100)	100	0.391

**Table 6:** Life table for endpoint all-cause revision

Interval	Number	Failures	Withdrawn	At risk	Failure rate (%)	Cumulative survival	95% CI
0	111	2	0	111	2	0.98	
1	109	0	0	109	0	0.98	
2	109	1	18	101	1	0.97	
3	91	0	24	79	0	0.97	
4	67	0	17	58	0	0.97	
5	50	0	50	25	0	0.97

Among SK baseplates, one in five implanted in varus (MPTA < 87°) required revision for mechanical failure of the tibia, and this was significant (*p* = 0.045, Fisher’s exact test). Three of five standard tibial baseplates implanted in varus with MPTA < 87° required return to theatre, with a relative risk of 9.9 (1.97–49.9 95% CI, *p* = 0.031, Fisher’s exact test) compared with other knees.

## Discussion

This study found no difference in early tibial component subsidence or loosening between standard tibial baseplates and UBP implants in patients with severe obesity when using a cemented cruciate retaining TKA. Similarly, joint-specific function, health-related quality of life, pain severity or pain location did not differ by type of implant. Rates of complications, 5-year survival and reoperations did not differ significantly between implants. The only case of tibial component mechanical failure occurred with a standard baseplate, albeit in varus alignment (MPTA < 87°). Varus alignment of the limb following TKA was associated with increased risk of return to theatre and revision across the entire cohort. Whether tibial implant geometry or alignment is most important in patients with severe obesity remains unclear.

Patients with a normal BMI have been shown to have comparable rates of aseptic loosening with both non-stemmed and stemmed tibial components [23]. The use of non-stemmed components is considered safe in this population and is utilised to reduce costs, shorten operative time and preserve bone-stock [23]. However, in patients with higher BMIs, some studies have demonstrated a benefit to using stemmed tibial components in terms of reduced revision rates [11–14]. Parratte et al. performed a randomised controlled trial of standard versus stemmed (10 × 100 mm stem) TKA in patients with BMI > 30 kg/m^2^, also stratifying a subgroup of BMI > 35 kg/m^2^ patients [24]. They reported a modest, clinically insignificant difference in Knee Injury and Osteoarthritis Outcome Score (KOOS) scores at 2 years in favour of stems, stating that they could not recommend the routine use of additional stems for obesity alone [24]. A retrospective study of TKA with or without a short additional 30 mm stem, found no difference between groups in terms of radiographic tibial failures [25]. Other studies in support of stems are retrospective, with some including implants of different designs [14, 26], relatively low BMI thresholds [14] or small sample sizes of patients with both elevated BMI and stems [13]. All involve adding stems to tibial components rather than investigating keeled and stemmed primary tibial baseplates.

Over 3 million Triathlon TKAs have been implanted worldwide to date, with 5- and 10-year studies of survival and PROMs published [27, 28]. To the authors knowledge, outcomes specific to the UBP have not previously been reported. Although no UBP implants were revised for mechanical failure, two were revised for infection. This may be related to the increased risk of periprosthetic joint infection in this severely obese patient group [29].

Cox et al. demonstrated that the most common mechanisms of TKA mechanical failure were failure of the cement–implant interface and tibial varus collapse, defined as a change in component position of > 10° [30]. They advocated for use of stemmed tibial implants in high-risk patients. Our findings similarly showed that degree of varus implantation (FTA < 177°or MPTA < 87°) was associated with increased complications, specifically lateral knee pain and an increased rate of revision. The one case of mechanical tibial failure in the current study occurred in a tibial component with varus malalignment and this, rather than tibial baseplate design, may have contributed to its early loosening. Using longer stems may actually aid alignment and it may be that the beneficial effect of stems in obese patients [12, 13, 25] is in optimising alignment rather than implant–bone integration. Using intramedullary referencing may help to reduce tibial alignment outliers. Therefore, caution should be used if moving away from mechanical alignment philosophies in severe obesity.

The current study reports early outcomes, with follow-up at a mean of 4.9 years (minimum 2.1 years). Aseptic loosening is most commonly observed as a late complication, with an average time from implantation to revision of 5.5 years (range 0.03–24.2 years) [6]. Although obesity is associated with an increased risk of early failure [3, 31], with 40% of aseptic loosening occurring in the first 2 years in these patients [26], longer term follow-up is required to detect differences between implants in the medium-to-longer term. Though longer-term follow-up is required, it is also important to look for and explore early failures.

Similar to Steere et al. [25], the current study demonstrated no difference in early tibial failures between implants. The sample size of 111 meant the study was under-powered to show statistical significance when comparing rare outcomes between implant groups. Only one patient (0.9%) in the study required reoperation for aseptic loosening, though this is at an early stage of follow-up (minimum 2 years, mean 4.9 years). Further cases of loosening or subsidence may occur with longer follow-up. Whether the theoretical mechanical advantages of a stemmed geometry translates into superior longer-term survival requires longer follow-up and a larger sample size. Though statistically it was not possible to demonstrate tibial baseplate design superiority, the tibial loosening rate shown by both implants compares favourably at this timepoint in this patient group to that reported by other studies: non-stemmed 71.4% versus stemmed 100% at 4 years [13]; non-stemmed 93.4% versus stemmed 100% at minimum 2 years [14]. Other limitations of this study include its retrospective nature. The use of the surgeon’s discretion presents room for bias in the study. Since there were no significant differences between patient groups in terms of demographic or comorbidities, it is uncertain what factors influenced the surgeons to choose each respective implant. It remains unclear whether tibial component geometry or alignment is a more important risk factor for tibial failure in obesity as varus alignment of the limb and of the tibial component were associated with increased risk of return to theatre and revision.

## Conclusions

This study suggests favourable early-to-mid-term outcomes for both standard keel and short stemmed tibial baseplate geometries as part of a cemented cruciate retaining TKA in patients with a BMI ≥ 40 kg/m^2^. Using this TKA system in severely obese patients, there was a single case of early tibial loosening, giving a 5-year Kaplan–Meier survival of 98.1% using the standard tibial baseplate and 100% using the universal baseplate.

## Data Availability

The datasets generated during and/or analysed during the current study are not publicly available due to patient confidentiality but are available from the corresponding author on reasonable request.
